# A rare case of central pancreatectomy for isolated complete pancreatic neck transection trauma

**DOI:** 10.1186/s12893-019-0557-x

**Published:** 2019-07-12

**Authors:** Bingqing Du, Xin Wang, Xing Wang, Shashi Shah, Nengwen Ke

**Affiliations:** Department of Pancreatic Surgery, West China Hospital, Sichuan University, No 37, Guo Xue Xiang, Chengdu, 610041 Sichuan China

**Keywords:** Pancreas, Trauma, Isolated pancreatic injury, Central pancreatectomy

## Abstract

**Background:**

Pancreatic trauma accounts for only 0.2% of blunt trauma and 1–12% of penetrating injuries. Injuries to other organs, such as spleen, liver, or kidney, are associated with 50.5% of the cases. The isolated complete traumatic transection of the pancreatic neck is rare. In the past, pancreatoduodenectomy or distal pancreatectomy and splenectomy was the standard care for patients with traumatic transection of the pancreatic head, duodenum or distal pancreas, and pancreatic neck. However, limited cases have been reported on the central pancreatectomy for pancreatic neck injuries. We present a rare case of a 21-year-old male patient who received central pancreatectomy for isolated complete traumatic transection of the pancreatic neck.

**Case presentation:**

A 21-year-old male patient with mild abdominal pain and showed no apparent abnormality in the initial abdominal computed tomography (CT) was brought to the local hospital’s emergency department due to a traffic accident. The patient’s abdominal pain became progressively worse during observation in the hospital that led to the patient being referred to our hospital. The patient’s vital signs were stable, and a physical examination revealed marked tenderness and rebound pain throughout the abdomen. The patient’s white blood cells were increased; The serum amylase and lipase levels were elevated. The abdominal computed tomography revealed pancreatic neck parenchymal discontinuity, peripancreatic effusion, and hemorrhage. The patient underwent exploratory laparotomy. Intraoperative examination identified the neck of the pancreas was completely ruptured, and no apparent abnormalities were observed in the other organs. The patient underwent central pancreatectomy and Roux -Y pancreaticojejunostomy. The patient was treated with antibiotics, acid inhibition and nutritional supports for 10 days after surgery. Symptoms of the patient were significantly relieved, and white blood cells, serum amylase, and lipase levels returned to normal. The patient underwent follow up examination for 6 months with no evidence of exocrine or endocrine insufficiency.

**Conclusions:**

Central pancreatectomy is an effective pancreas parenchyma preserving procedure, may be a promising alternative to distal pancreatectomy and splenectomy for this complex pancreatic trauma in hemodynamically stable patients. Patient selection and surgeon experience are crucial in the technical aspects of this procedure.

## Background

Pancreatic trauma accounts for only 0.2% of blunt trauma and 1–12% of penetrating injuries, which can be associated with morbidity and mortality rates of 30–62% and 10–30%, respectively [1]. Pancreatic injuries comprise 17.3, 9.6, and 22.6% of the head of the pancreas, pancreatic body, and pancreatic tail, respectively. Injuries to other organs, such as spleen, liver, or kidney, are associated with 50.5% of the cases [1, 2]. The isolated complete traumatic transection of the pancreatic neck is rare. The choice of surgical procedures generally depends on the site of the pancreatic injury. In the past, pancreatoduodenectomy or distal pancreatectomy and splenectomy was the standard care for patients with traumatic transection of the pancreatic head, duodenum or distal pancreas, and pancreatic neck [1]. However, limited cases have been reported on the central pancreatectomy (CP) for pancreatic neck injuries. In this study, we present a rare case of a 21-year-old male patient who received CP for isolated complete traumatic transection of the pancreatic neck.

## Case presentation

**Fig. 1:**
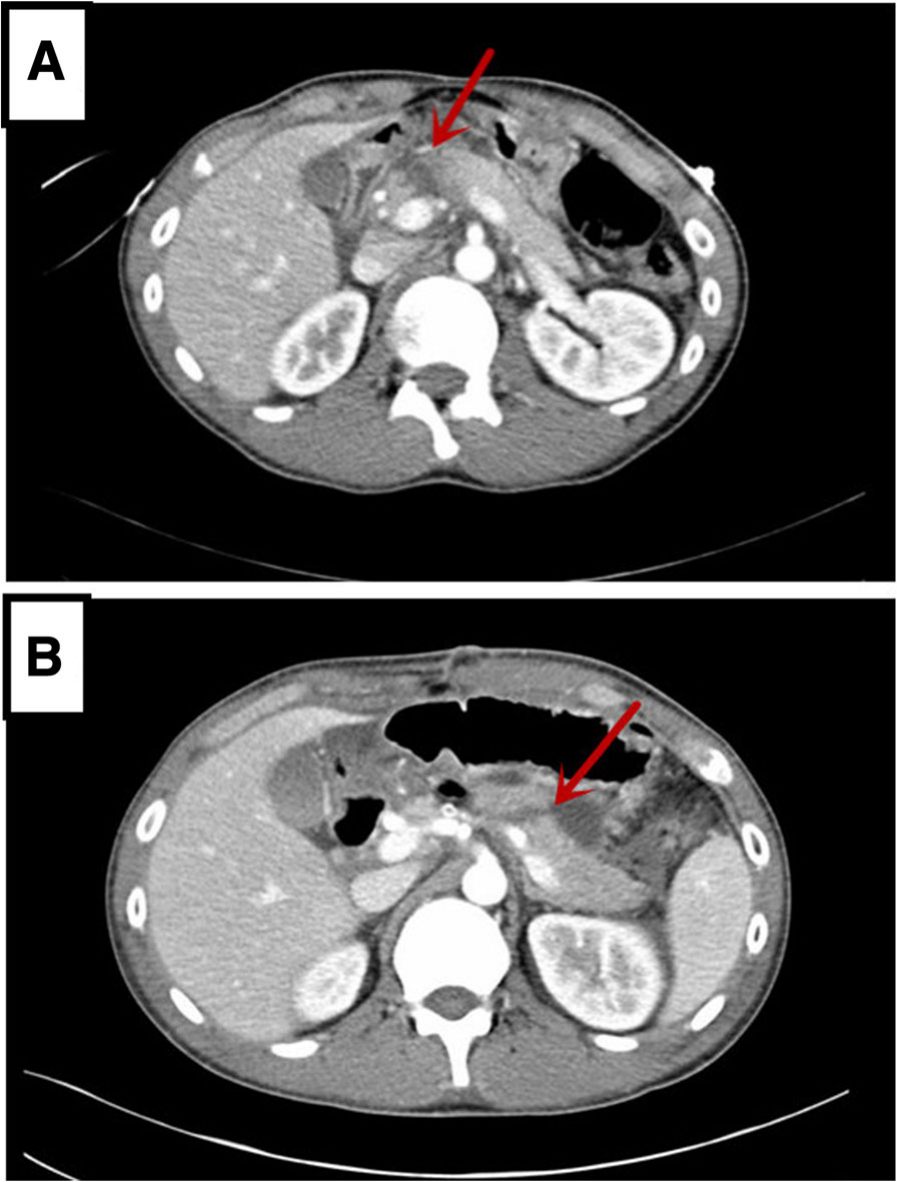
(**a**) Abdominal computed tomography (CT) reveal discontinuous pancreatic neck parenchyma, peripancreatic effusion, and hemorrhage (the red arrow indicates the pancreatic neck complete transection). (**b**) Postoperative abdominal CT show a small amount of gas and fluid in the abdominal cavity, and the anastomotic site of the pancreas and intestine healed well (the red arrow denotes the anastomotic site of the pancreas and intestine)

**Fig. 2:**
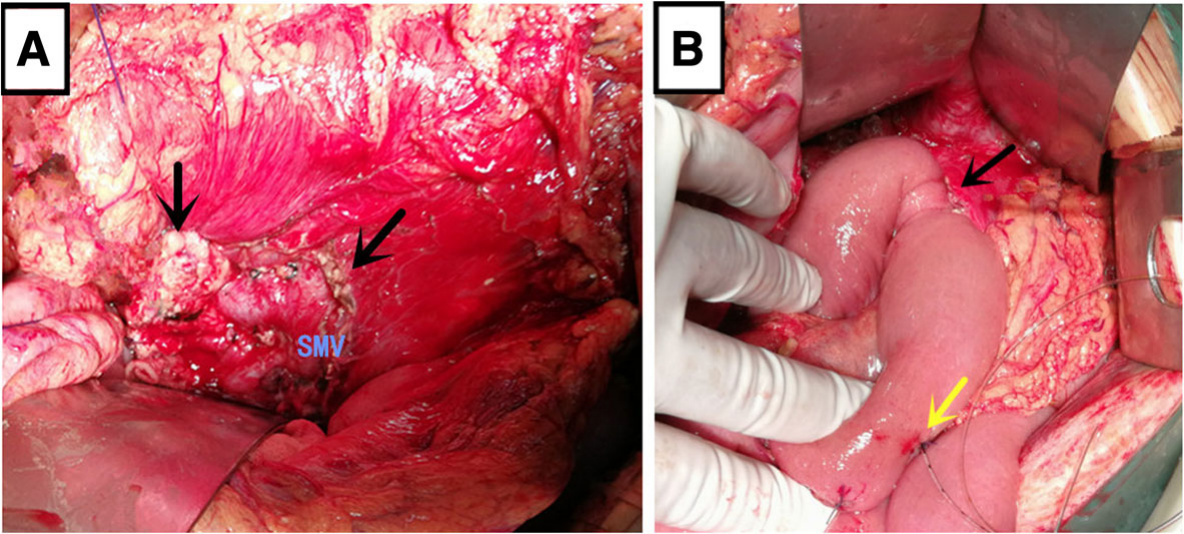
Patient underwent central pancreatectomy. (**a**) We removed the damaged pancreas around the transection of pancreatic neck until normal pancreatic tissue was exposed (the black arrow denotes the normal pancreatic tissue after removal of damaged pancreas around the transection of the pancreatic neck). (**b**) Reconstruction of the distal pancreatic remnant was accomplished by using Roux-Y pancreaticojejunostomy (the black arrow depicts the anastomotic site of the pancreas and intestine, whereas the yellow arrow shows the external drainage stent)

**Fig. 3:**
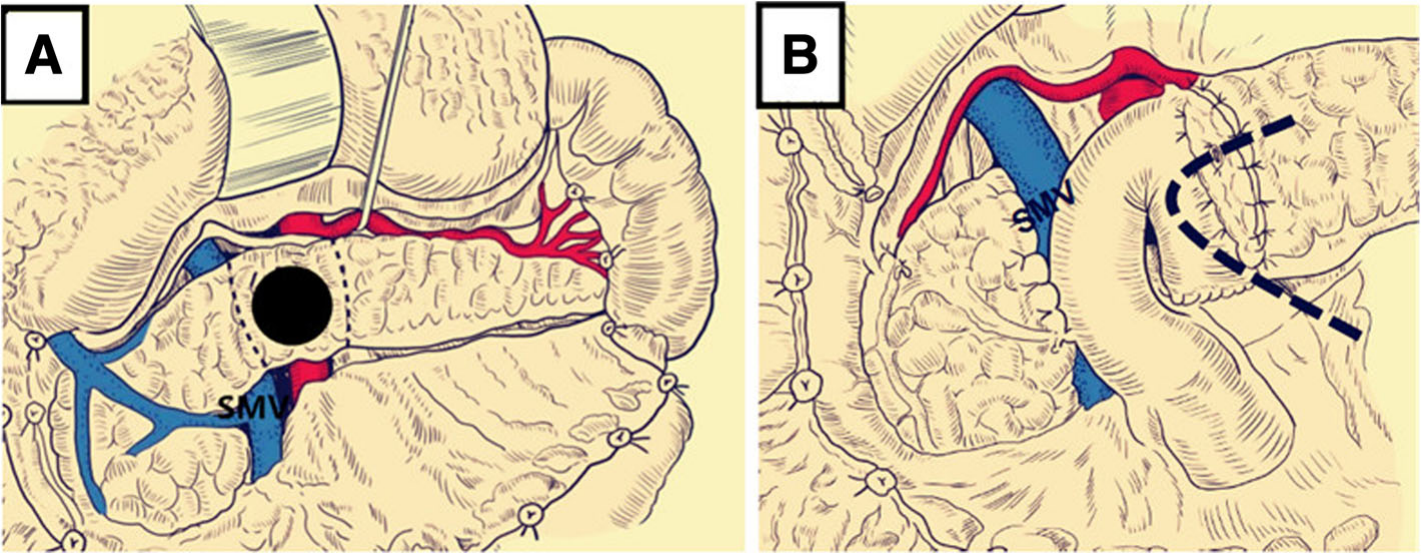
Surgical principle of CP. (**a**) We removed the damaged pancreatic tissue around the transection of the pancreatic neck (the black-dotted line shows the reaction area of the pancreas). (**b**) Oversewed the proximal pancreatic head with interrupted absorbable sutures, anastomosis of pancreatic duct and jejunal mucosa, and the external drainage stent was placed at the anastomosic site of the pancreas and intestine (the black-dotted line illustrates the placement of the external drainage stent)

A 21-year-old male patient with mild abdominal pain and showed no apparent abnormality in the initial abdominal computed tomography (CT) was brought to the local hospital’s emergency department due to a traffic accident. The patient’s abdominal pain became progressively worse during observation in the hospital that led to the patient being referred to our hospital. The patient’s vital signs were stable, and a physical examination revealed marked tenderness and rebound pain throughout the abdomen. The patient’s white blood cells were increased (11.64 × 10^9^/L, normal range: 4–9 × 10^9^/L). The serum amylase and lipase levels were elevated (1,707 and 1,160 IU/L, normal range: 25–125 and 13–60 IU/L, respectively). The abdominal computed tomography revealed pancreatic neck parenchymal discontinuity, peripancreatic effusion, and hemorrhage (Fig. 1a). Then, the patient underwent exploratory laparotomy. Intraoperative examination identified a large amount of flaxen ascites in the abdominal cavity, the neck of the pancreas was completely ruptured, the head and tail of the pancreas were congested and edematous with accompanying black necrotic tissue of the pancreas, scattered saponification spots were observed in the greater omentum, and no apparent abnormalities were observed in the other organs. The patient underwent central pancreatectomy (CP). We removed the damaged pancreas around the transection of the pancreatic neck until the normal pancreatic tissue was exposed (Fig. 2a), reconstruction of the distal pancreatic remnant was accomplished by Roux-Y pancreaticojejunostomy (Fig. 2b). The surgical principle can be referenced by Fig. 3 or described by Motoi F et al. [13]. The patient was treated with antibiotics, acid inhibition, and nutritional support for 10 days after surgery. Symptoms of the patient were significantly relieved, and white blood cells, serum amylase, and lipase levels returned to normal. Postoperative abdominal CT showed a small amount of gas and fluid in the abdominal cavity, whereas the anastomotic site of the pancreas and intestine healed well (Fig. 1b). The patient underwent follow up examination for 6 months with no evidence of exocrine or endocrine insufficiency.

## Discussion and conclusions

Trauma to the pancreas only occurs in 0.2–2% of all trauma patients, and 3–12% of patients with abdominal injury are due to the retroperitoneal location of the pancreas [3]. The majority of injury occurrence is primarily related to other associated organs, such as the liver, major vascular structures, colon or small bowel, duodenum, stomach, spleen, and kidney [3]. Isolated pancreatic injury refers to the abdominal trauma when the pancreas is the only organ injured, and its occurrence is less than 1% [4]. The occurrence of complete pancreatic transection in isolated pancreatic injuries is limited, and the majority of cases occur in young males with blunt abdominal trauma, such as vehicular accidents, as a result of impact with the steering wheel that produces direct compression of the pancreas against the lumbar vertebrae and may cause pancreatic contusion, hematoma, transection of the gland, and rupture of the pancreatic duct in the line of the superior mesenteric vein at the neck of the gland [4, 5]. The diagnosis of isolated pancreatic injury is difficult or delayed because the retroperitoneal site of the pancreas can mask significant clinical findings, even in the presence of pancreatic duct disruption [6]. Elevated serum amylase is an important diagnostic indicator. High-quality CT scans, which have both sensitivity and specificity of up to 80%, represent the best noninvasive diagnostic method for the detection of pancreatic injuries [6, 7]. Elevated serum amylase and typical CT images prompt the diagnosis of isolated pancreatic neck injuries. However, initial CT results may be negative with pancreatic injury in certain cases. In our case, the patient’s abdominal pain was minimal at first, and the abdominal CT result was negative. Then, the patient’s abdominal pain became progressively worse that led to his being transferred to our hospital. The isolated pancreatic injury was determined in the CT scan performed at our hospital. Delays are associated with increasing morbidity and mortality in isolated pancreatic injuries; therefore, a follow-up CT scan may be important to make a definitive diagnosis [8].

The integrity of the main pancreatic duct (MPD) is an important determinant for isolated pancreatic trauma. For patients with MPD injury or complete transection, surgery is still recommended [9]. Distal pancreatectomy and/or splenectomy were the previous standard care for patients with isolated traumatic transection of the pancreatic neck [10, 11] likely because distal pancreatectomy is less time consuming than CP in trauma patients where time is essential; in addition, surgeon experience is of utmost importance in the technical aspects of CP [12]. CP is suitable for hemodynamically stable patients and must only be performed at high-volume centers by experienced surgeons. Laparoscopic central pancreatectomy is time consuming, which ofen used in the elective surgery and is not recommended in an emergency situation. In our case, the patient was young and his vital signs were stable, and the surgeons were highly qualified pancreatic doctors. The preservation of body tissue and tail of the pancreas has increased proportions of insulin cells and can reduce postoperative exocrine and endocrine insufficiencies [1]; hence, we performed CP and Roux-Y pancreaticoenterostomy [13]. The rate of new onset diabetes mellitus was 4.7% versus 35% (*p* = 0.0129) in patients with CP compared with distal pancreatectomy group. The body weight in the distal pancreatectomy group was significantly lower than that in the CP group after 1 and 2 years of surgery (1 year, *P* < 0.0001; 2 years, *P* = 0.0055), and the body weight of patients undergoing CP was better than the preoperative values within 2 years after surgery [14]. In our case, the patient underwent follow-up examination for 6 months, and no evidence of insufficient endocrine and exocrine functions were observed. In addition, pancreatic leakage is a common complication of CP [15]. In our opinion, the prevention of pancreatic leakage should completely remove the inactivated and damaged inflammatory tissues of the body and head of the pancreas to normal tissue, anastomosis of the pancreatic duct and jejunal mucosa, the pancreas stump interlocked with the U-shaped suture, external drainage or internal drainage stent placed at the anastomosis of pancreas and intestine, two drainage tubes and one irrigation tube were placed at the pancreaticojejunostomy and the stump of the head of pancreas. In our case, the serum amylase value in the abdominal drainage tube was more than three times higher than the normal value 3 days after the operation that recovered to the normal value 20 days after surgery.

In conclusion, we successfully applied CP, which is a rare surgical strategy in rare emergency cases of complete pancreatic neck transection in isolated pancreatic trauma. CP is an effective pancreas parenchyma preserving procedure that may be a promising alternative to distal pancreatectomy and splenectomy for complex pancreatic trauma in hemodynamically stable patients. Patient selection and surgeon experience are crucial in the technical aspects of this procedure.

## Data Availability

All the data supporting our findings are contained within the manuscript.

## Abbreviations

CP: Central pancreatectomy
MPD: Main pancreatic duct
SMV: Superior mesenteric vein
